# Low density lipoprotein receptors LRP-1 and LRP-2 in C. elegans

**DOI:** 10.17912/micropub.biology.000154

**Published:** 2019-08-27

**Authors:** Paul J Minor, Paul W Sternberg

**Affiliations:** 1 Division of Biology and Biological Engineering, Caltech, Pasadena, CA 91125; 2 Department of Biology, Hopkins Marine Station of Stanford University, Pacific Grove, CA 93950

**Figure 1. LRP-2 domains and phylogeny:**
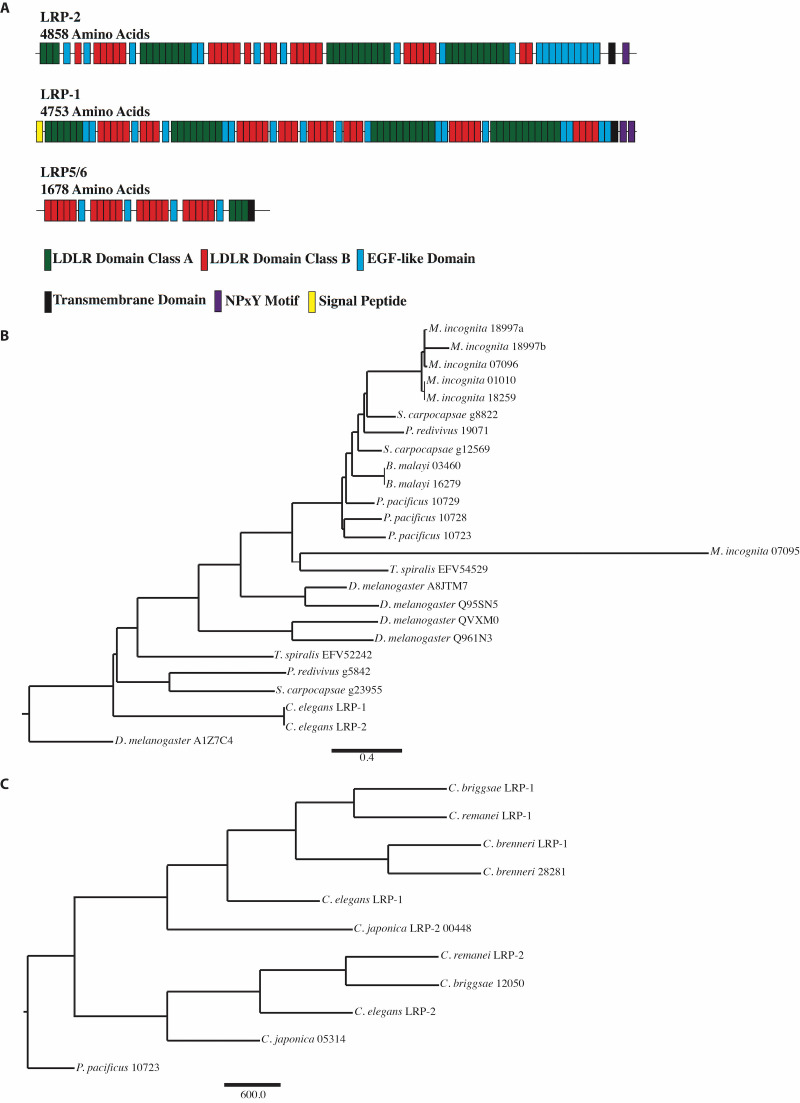
(A) Protein domains of LRP-2, LRP-1, and *Drosophila* Arrow. *C. elegans* does not possess a true ortholog of Arrow (LRP5/6); however, it does possess multiple megalin-like proteins that contain LDLR Class A repeats, LRDR Class B repeats, and EGF-like domains that are found in varieties of low density like lipoprotein receptors such as megalin and Arrow. All domains are color-coded and drawn to approximate scale according to the SMART database. (B) An evolutionary tree based on the protein sequence of LRP-1 and LRP-2 in nematodes and megalin in *Drosophila melanogaster*. Based on sequence similarity, position in the genome, and clustering, it appears that LRP-2 is the result of a recent duplication in *Caenorhabditis*. (C) Within *Caenorhabditis*, LRP-1 orthologs cluster together and LRP-2 orthologs cluster. *Pristionchus pacificus* is used as the outgroup.

## Description

The regulation of vulval cell lineage polarity is controlled by Wnt signaling. Previously known components involved in the regulation of vulval cell lineage polarity include LIN-17, LIN-18, CAM-1, and VANG-1 (Inoue *et al*., 2004; Gleason *et al*., 2006; Green *et al*., 2008). A directed bioinformatics screen of known Wnt pathway components was performed to find additional genes involved in directing vulval orientation. A BLAST was run using other known Wnt receptors and it was determined that *C. elegans* does not contain a true ortholog of *Drosophila* LRP5/6 (Arrow) (He *et al*., 2004; Eisenmann, 2005), but does have multiple low-density lipoprotein receptors, including LRP-1 and LRP-2 (Figure 1). Like other low-density lipoprotein receptors, both LRP-1 and LRP-2 contain many LDLR Domain Class A and Class B repeats, EGF-like domains, and a transmembrane domain. However, having approximately three times as many amino acids, LRP-1 and LRP-2 are more similar to megalin than LRP5/6 (Yochem *et al*., 1999). The absence of LRP5/6 within *C. elegans* but presence in flies and all other higher order organisms suggests that the gene encoding LRP5/6 arose after nematodes, potentially from either LRP1 or LRP2/megalin, as both receptors contain the entire extracellular portion of LRP5/6 in a single contiguous sequence block (Figure 1).

Our examination of the protein sequence of LRP-1 and LRP-2 indicates that most nematodes have at least two copies of LRP-like proteins with *C. elegans* LRP-1 and LRP-2 being highly similar possibly due to a recent duplication and divergence (Figure 2). Comparing the sequences across *Caenorhabditis* we find that LRP-1 proteins cluster together and LRP-2 proteins also form their own cluster. Based on location in the genome and sequence similarity from protein alignment, we believe that *Caenorhabditis lrp-2* is a recent duplication and divergence of *lrp-1* (Figure 2).

## Methods

Available predicted protein datasets from nematodes were obtained from WormBase release WS225 (www.wormbase.org). Other sequences were obtained from the NCBI/NIH repository (ftp://ftp.ncbi.nih.gov/genomes). Maximum likelihood (ML) analyses with 1,000 bootstraps were done using the RAxML BlackBox server (http://phylobench.vital-it.ch/raxml-bb). Protein domain analysis performed using the SMART protein domain analysis website (http://smart.embl-heidelberg.de)
